# Immune Checkpoint Inhibitor-Induced Cerebral Pseudoprogression: Patterns and Categorization

**DOI:** 10.3389/fimmu.2021.798811

**Published:** 2022-01-03

**Authors:** Hans Urban, Eike Steidl, Elke Hattingen, Katharina Filipski, Markus Meissner, Martin Sebastian, Agnes Koch, Adam Strzelczyk, Marie-Thérèse Forster, Peter Baumgarten, Michael W. Ronellenfitsch, Joachim P. Steinbach, Martin Voss

**Affiliations:** ^1^ Dr. Senckenberg Institute of Neurooncology, University Hospital Frankfurt, Goethe University, Frankfurt/Main, Germany; ^2^ University Cancer Center Frankfurt (UCT), University Hospital Frankfurt, Goethe University, Frankfurt/Main, Germany; ^3^ German Cancer Consortium (DKTK), Partner Site Frankfurt/Mainz; and German Cancer Research Center (DKFZ), Heidelberg, Germany; ^4^ Frankfurt Cancer Institute (FCI), Georg-Speyer-Haus, Frankfurt/Main, Germany; ^5^ Institute of Neuroradiology, University Hospital Frankfurt, Goethe University, Frankfurt/Main, Germany; ^6^ Institute of Neurology (Edinger Institute), University Hospital Frankfurt, Goethe University, Frankfurt/Main, Germany; ^7^ Department of Dermatology, Venereology and Allergology, University Hospital Frankfurt, Goethe University, Frankfurt/Main, Germany; ^8^ Department of Medicine II, Hematology/Oncology, University Hospital Frankfurt, Goethe University, Frankfurt/Main, Germany; ^9^ Department of Thoracic Surgery, Agaplesion Markuskrankenhaus, Frankfurt/Main, Germany; ^10^ Epilepsy Center Frankfurt Rhine-Main and Department of Neurology, Goethe-University, Frankfurt am Main, Germany; ^11^ LOEWE Center for Personalized Translational Epilepsy Research (Cepter), Goethe-University Frankfurt, Frankfurt am Main, Germany; ^12^ Department of Neurosurgery, University Hospital Frankfurt, Goethe University, Frankfurt/Main, Germany

**Keywords:** immune checkpoint inhibitors (ICI), immunotherapy, cerebral pseudoprogression, immune related adverse events (irAE), brain metastases, neurological side effects, neurological complication

## Abstract

**Background:**

The inclusion of immune checkpoint inhibitors (ICIs) in therapeutic algorithms has led to significant survival benefits in patients with various metastatic cancers. Concurrently, an increasing number of neurological immune related adverse events (IRAE) has been observed. In this retrospective analysis, we examine the ICI-induced incidence of cerebral pseudoprogression and propose a classification system.

**Methods:**

We screened our hospital information system to identify patients with any in-house ICI treatment for any tumor disease during the years 2007-2019. All patients with cerebral MR imaging (cMRI) of sufficient diagnostic quality were included. cMRIs were retrospectively analyzed according to immunotherapy response assessment for neuro-oncology (iRANO) criteria.

**Results:**

We identified 12 cases of cerebral pseudoprogression in 123 patients treated with ICIs and sufficient MRI. These patients were receiving ICI therapy for lung cancer (n=5), malignant melanoma (n=4), glioblastoma (n=1), hepatocellular carcinoma (n=1) or lymphoma (n=1) when cerebral pseudoprogression was detected. Median time from the start of ICI treatment to pseudoprogression was 5 months. All but one patient developed neurological symptoms. Three different patterns of cerebral pseudoprogression could be distinguished: new or increasing contrast-enhancing lesions, new or increasing T2 predominant lesions and cerebral vasculitis type pattern.

**Conclusion:**

Cerebral pseudoprogression followed three distinct patterns and was detectable in 3.2% of all patients during ICI treatment and in 9.75% of the patients with sufficient brain imaging follow up. The fact that all but one of the affected patients developed neurological symptoms, which would be classified as progressive disease according to iRANO criteria, mandates vigilance in the diagnosis and treatment of ICI-induced cerebral lesions.

## Introduction

Cancer cells can suppress immune system activation by hijacking inhibitory pathways of T cell activation. Major elements of these inhibitory checkpoints are cytotoxic T-lymphocyte-associated antigen 4 (CTLA-4), programmed cell-death protein 1 (PD-1) and its ligand PD-L1. Immune-checkpoint inhibitors (ICIs) potently suppress these inhibitory pathways, thereby disinhibiting antitumor immune responses. The efficacy of ICIs has been demonstrated across several cancers including advanced malignant melanoma (1, 2) and non-small cell lung cancer (NSCLC) (3).

Target lesion pseudoprogression associated with ICIs is a well-established phenomenon for NSCLC and melanoma and is caused by an infiltration of immune cells and inflammation prior to tumor shrinkage (4). An extracranial pseudoprogression rate of 7% has been reported in the KEYNOTE-001 trial of pembrolizumab (PD-1 inhibitor) for advanced melanoma (5). The efficacy of ICIs has been demonstrated for treating cerebral metastases as well, both as monotherapy and in combination with radiation therapy (6, 7). In these patients, it is of major importance to detect immune-related adverse events (IRAE), which can only be differentiated from progressive disease by specific additional examinations (8). Until now, only single case reports or case series of cerebral pseudoprogression have been published and the incidence of cerebral pseudoprogression after ICI treatment is unknown. Imaging patterns of cerebral pseudoprogression reported so far are diverse and include an increase in MRI contrast enhancement in metastatic lesions with an increase of adjacent edema (9–11), small dotted cerebral bleedings (12) and new FLAIR hyperintense lesions (13) distant from cerebral metastases. The FLAIR hyperintense lesions have been interpreted as inflammatory central nervous system (CNS) demyelination in one case (14) and as immune-mediated cerebellitis in another case (15).

A systematic evaluation of frequency and patterns of cerebral pseudoprogression in a larger cohort has not yet been reported. Also, it is unclear whether the onset of pseudoprogression is influenced by co-factors such as prior radiation therapy or the presence of brain metastases. Given that immune therapy with chimeric-antigen-receptor T-cells (CAR-T) can cause severe cerebral neurotoxicity (immune effector cell-associated neurotoxicity syndrome; ICANS), cerebral pseudoprogression independent of brain metastases or primary brain cancer seems possible (16). To address these issues, we performed a retrospective analysis of all patients with available cerebral MRI who received ICI treatment at our hospital regardless of tumor histology and presence or absence of cerebral metastatic disease.

## Materials and Methods

**Figure 1:**
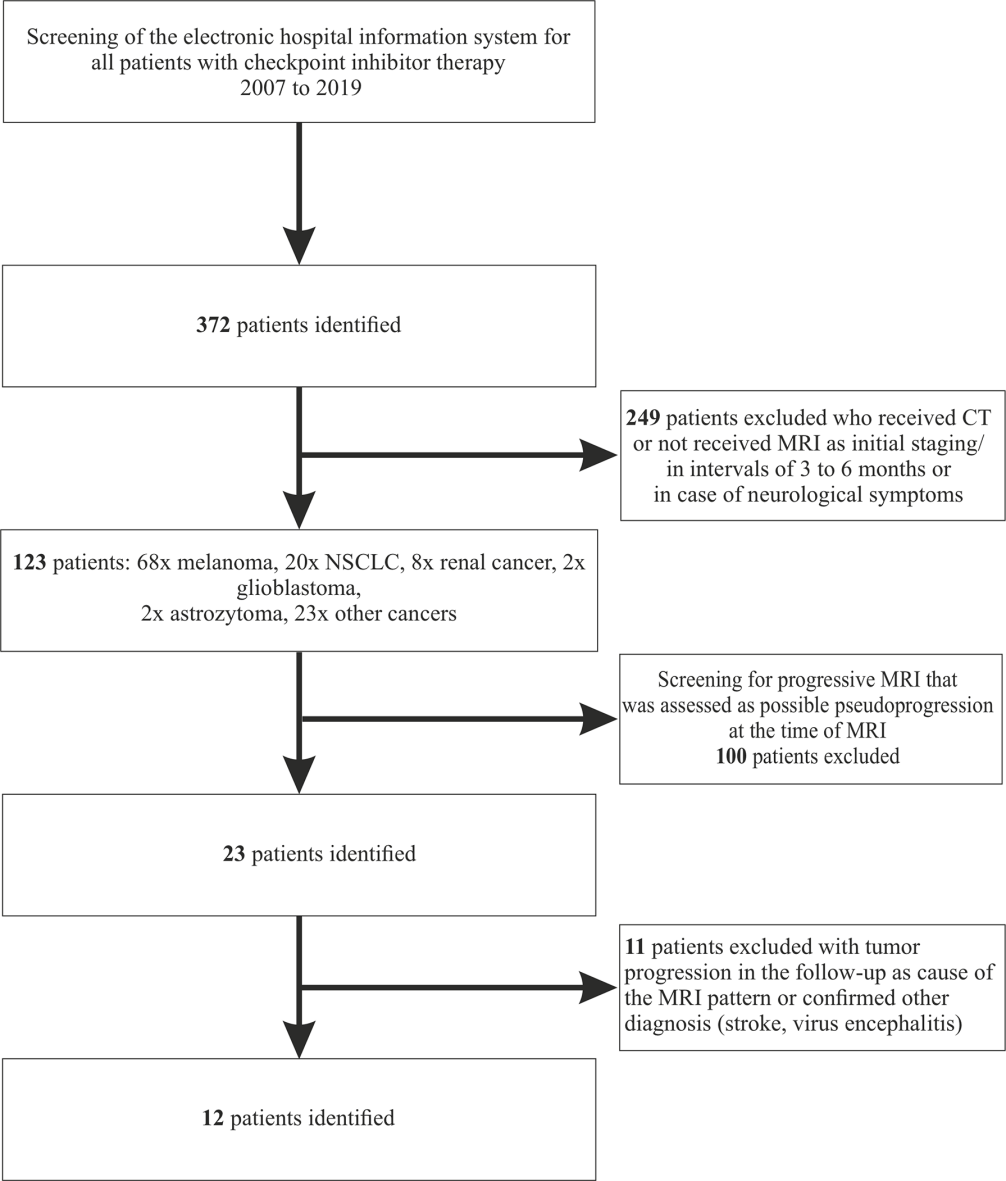
Consort Flow-diagram.

We performed a retrospective analysis of patients treated in our hospital between the years 2007 and 2019 to identify patients with any in-house ICI treatment (pembrolizumab, ipilimumab, nivolumab, atezolizumab, avelumab) for any tumor disease (**Figure 1**). Since cerebral metastasis had been a contraindication for ICIs in the initial pivotal studies, all identified patients had received a cCT or cMRI scan before starting treatment (17). Further MRI controls had either been scheduled at intervals of 3 to 6 months or were only performed when neurological symptoms occurred. Requirement for inclusion was the availability of a follow-up cMRI at least 3 months after initiation of checkpoint inhibitor therapy. Patients without MRI scan or with only CT scans in the follow-up were excluded. All MRIs were performed on 1.5 or 3 T scanners acquiring at least T1-weighted sequences with and without contrast agent, T2-weighted sequences [T2-turbo spin echo (T2-TSE) and fluid-attenuated inversion recovery (FLAIR)]. The MRIs were analyzed for progression by an experienced, board-certified neuroradiologist (ES, EH) (18). Patients with confirmed tumor progression in the next follow-up or other defined diseases causing the change in MRI, i.e. ischemic stroke or viral encephalitis, were then excluded based on immunotherapy response assessment in neuro-oncology (iRANO) (19). Only the image findings were used for the first screening. The iRANO criterion of significant clinical decline was not taken into account during this phase of data collection.

Microsoft Excel and GraphPad Prism 8.0.2 were used for data management and statistical analysis. Corel Draw 2019 was used to create figures.

Ethics approval for this retrospective data collection was obtained from the ethics committee of the University Hospital Frankfurt; Goethe University, Germany (Protocol-Number: SNO-13-2019).

## Results

### Patient Characteristics Prior to Diagnosis of Cerebral Pseudoprogression

We identified 12 patients with cerebral pseudoprogression in a cohort of 372 patients with ICI treatment (123 with sufficient brain imaging; 9.8% rate of cerebral pseudoprogression of the patients with sufficient imaging during the treatment) (**Figure 1**: Consort diagram). Mean age was 61 years (range 44 – 76 years), with a male predominance (75%). Primary tumor disease included lung cancer (n=5, NSCLC=4, SCLC=1), malignant melanoma (n=4), glioblastoma (n=1), hepatocellular carcinoma (n=1) and lymphoma (n=1). At the time of diagnosis of pseudoprogression, 10 patients had already been diagnosed with brain metastases or, in the one case of glioblastoma, primary cancer of the brain. 11 of 12 patients showed abnormal findings in the neurological examination upon diagnosis of pseudoprogression. In 6 of 12 patients, pseudoprogression manifested as a first occurrence of epileptic seizure or as a worsening of a known structural epilepsy. Details on EEG findings and treatment with antiseizure medication have previously been published (20). Three patients showed a paresis of arms or legs. Two patients presented with personality changes. Two patients had symptoms of increased intracranial pressure. One patient developed hypoacusis, one developed facial nerve palsy. In one patient, pseudoprogression was an incidental finding during a routine MRI and initially, no neurological deficit was present.

Notably, half of the patients with cerebral pseudoprogression had already experienced another type of IRAE during ICI treatment. Two patients had already suffered from autoimmune hepatitis before the onset of cerebral pseudoprogression. In one of these 2 patients, therapy was changed from ipilimumab/nivolumab to pembrolizumab. Additionally, 2 other patients had previously developed autoimmune-related hypophysitis, one patient had suffered from pneumonitis and one from dermatitis. All six patients had received prednisolone to treat these adverse drug reactions.

All patients were still under active ICI treatment upon diagnosis of cerebral pseudoprogression. Six patients were treated with pembrolizumab, 4 patients received a combination of nivolumab and ipilimumab, one patient was treated with atezolizumab and one with nivolumab. All treatments were administered in the respective standard ICI doses. Median time from the start of ICI therapy to diagnosis of cerebral pseudoprogression was 5 months (range 1-19 months). Seven patients had been treated exclusively with first-line nivolumab/ipilimumab or pembrolizumab. One patient had previously received a tumor specific immunotherapy by vaccination. The remaining 4 patients had received various pre-treatments with non-immune therapies (**Table 1**).

**Table 1:** Patient characteristics.

**Number of patients**	12
**Age at diagnosis of pseudoprogression [years]**	
Median (range)	61 (44 – 76)
**Sex**	
male	75.0% (9)
female	25.0% (3)
**Histology**	
NSCLC	33.3% (4)
SCLC	8.3% (1)
Melanoma	33.3% (4)
Glioblastoma	8.3% (1)
Lymphoma	8.3% (1)
Hepatocellular carcinoma	8.3% (1)
**Prior radiation therapy**	
None	25.0% (3)
Radiosurgery	41.6% (5)
Fractionated radiation therapy	33.3% (4)
**Checkpoint inhibitor therapy**	
Nivolumab	8.3% (1)
Nivolumab + Ipilimumab	33.3% (4)
Pembrolizumab	50.0% (6)
Atezolizumab	8.3% (1)
Prior other checkpoint inhibitor therapy	8.3% (1)
**Time from start of therapy** **to diagnosis of pseudoprogression [months]**	
Median (range)	5 (1 – 10)
**Other autoimmune adverse events**	50% (6)
Colitis	16.6% (2)
Hypophysitis	16.6% (2)
Pneumonitis	8.3% (1)
Dermatitis	8.3% (1)
MRI pattern*	N=15
Increased contrast-enhancement	33.3% (5)
Increased T2 lesions	40.0% (6)
Contrast-enhancement of cranial nerves	13.3% (2)
Vasculitis	13.3% (2)

*Multiple options to classify MRI findings are possible, if applicable.

Nine patients had been treated with radiation therapy directly after the diagnosis of brain metastasis (4 with fractionated radiotherapy and 5 with radiosurgery) and before diagnosis of cerebral pseudoprogression. None had been treated with whole brain radiation therapy. Three patients (with lymphoma, hepatocellular carcinoma and melanoma) had not received any cerebral radiation as part of their treatment. Median time between the end of radiotherapy and the diagnosis of pseudoprogression was 6 months (range 1 – 24 months)

### Increases in Contrast-Enhancing Lesions as a Type of ICI-Mediated Pseudoprogression

Three distinct patterns of cerebral pseudoprogression were detected in our patient collective. Six patients had an increase in contrast enhancement of preexisting lesions or presented with new contrast-enhancing lesions in the T1-weighted sequences after intravenous administration of contrast agent.

Progressive contrast enhancement could also present as an intraparenchymal lesion adjacent to the previous tumor manifestation within the irradiation field (1 patient) or distant to the initial tumor manifestation and outside the irradiation field (3 patients). In 2 of the 3 patients in whose pseudoprogression occurred outside the prior irradiation field, contrast enhancement of the cranial nerves was present.

**Figure 2:**
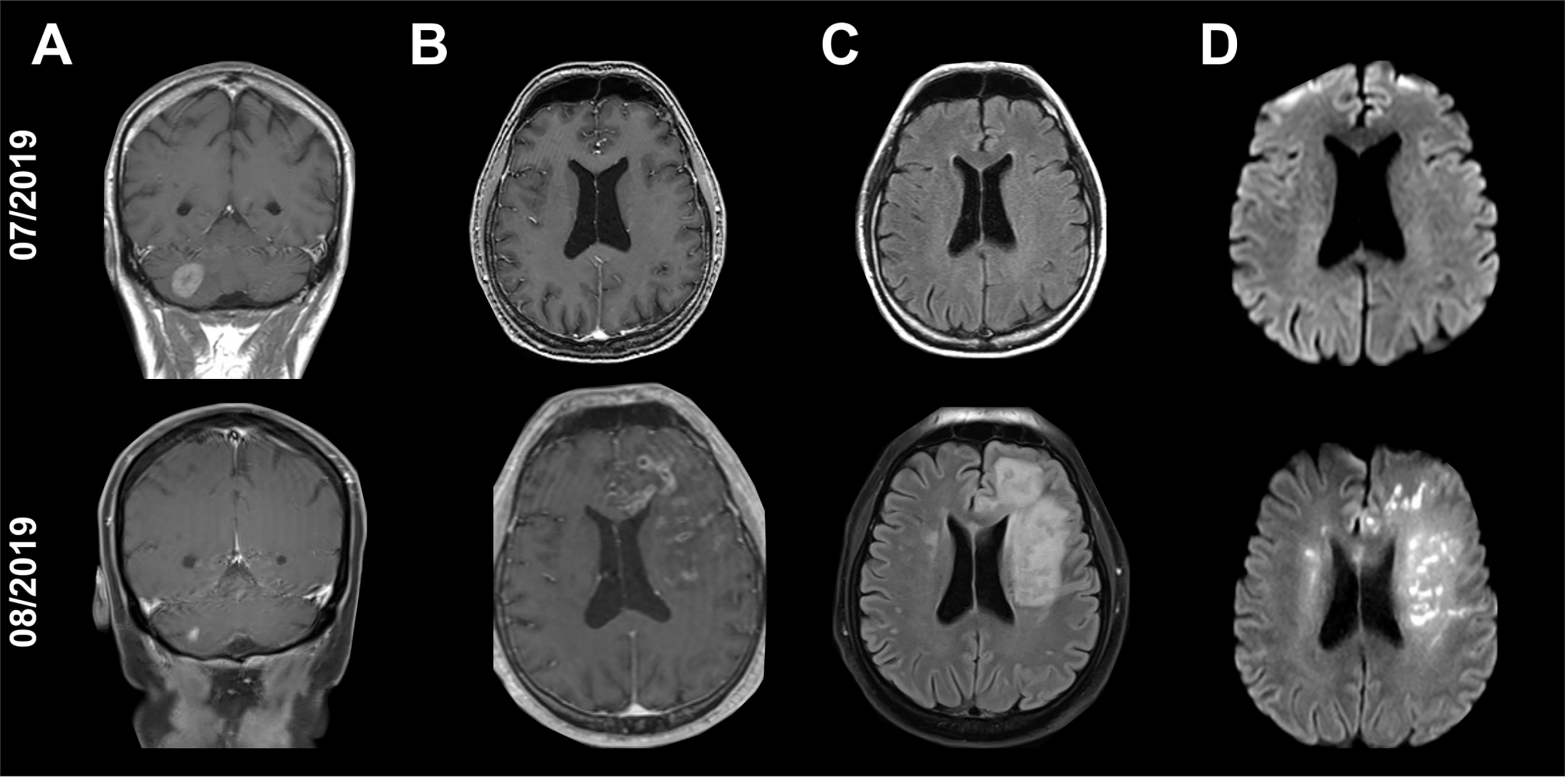
Cranial MRI scans of a 54-year-old patient with single, cerebellar metastasis of non-small-cell lung carcinoma. The MRI scan shows an excellent response of the cerebellar metastasis to the radiation therapy [**(A)**: T1-weighted, contrast enhanced images]. At the same time, new tubular contrast enhancements with adjacent edema and diffusion restrictions have appeared in the left frontal lobe distant to the irradiated cerebellar metastasis [**(B)**: T1-weighted, contrast enhanced images, **(C)**: Fluid-attenuated inversion recovery (FLAIR), **(D)**: Diffusion-weighted images (DWI, b1000)].

**Figure 3:**
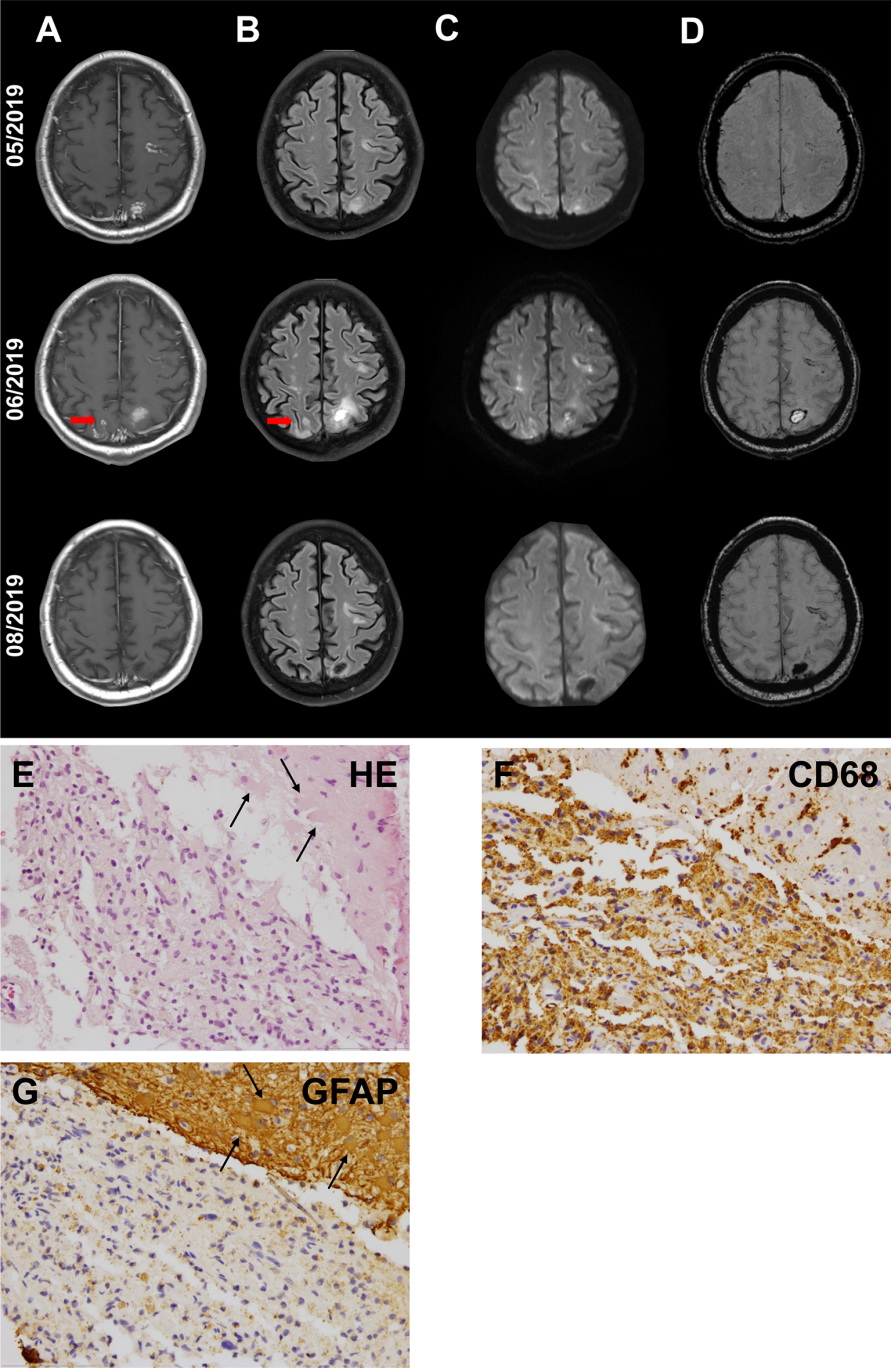
Cranial MRI scans of a 61-year-old patient with Hodgkin’s lymphoma in the thoracic and abdominal lymph nodes. Recurrent lymphoma had been treated with pembrolizumab since 12/2018. Initial CT scan of the brain as part of a whole body FDG-PET scan had shown no cerebral manifestations of the lymphoma (not shown). The patient had no neurological symptoms at the start of ICI therapy. The patient was admitted in 05/2019 with dizziness and nausea. First MRI (upper row) showed small nodular, cortical contrast enhancement **(A)** with corresponding hyperintense signal in fluid-attenuated inversion recovery (FLAIR) imaging **(B)** and diffusion-weighted imaging (DWI, b1000) (Arrows mark the biopsy site), **(C)** as well as small bleedings in susceptibility-weighted imaging (SWI) **(D)**. Cerebrospinal fluid was not indicative for cerebral lymphoma or bacterial or viral encephalitis. Histological evaluation of biopsy samples **(E–G)** revealed neither cerebral lymphoma nor JC-virus, but reactive CNS alterations with astrogliosis [**(E)**+**(G)**, arrows: astrocytes with reactive changes] and macrophage clearance **(F)**. First follow-up MRI after the discontinuation of pembrolizumab (middle row) showed a further progression of the lesion. Treatment with high-dose methylprednisolone and tapering dose of prednisolone in combination with everolimus was administered. First control under the immunosuppressive treatment (lower row) showed an improvement with regressive contrast enhancement.

**Figure 4:**
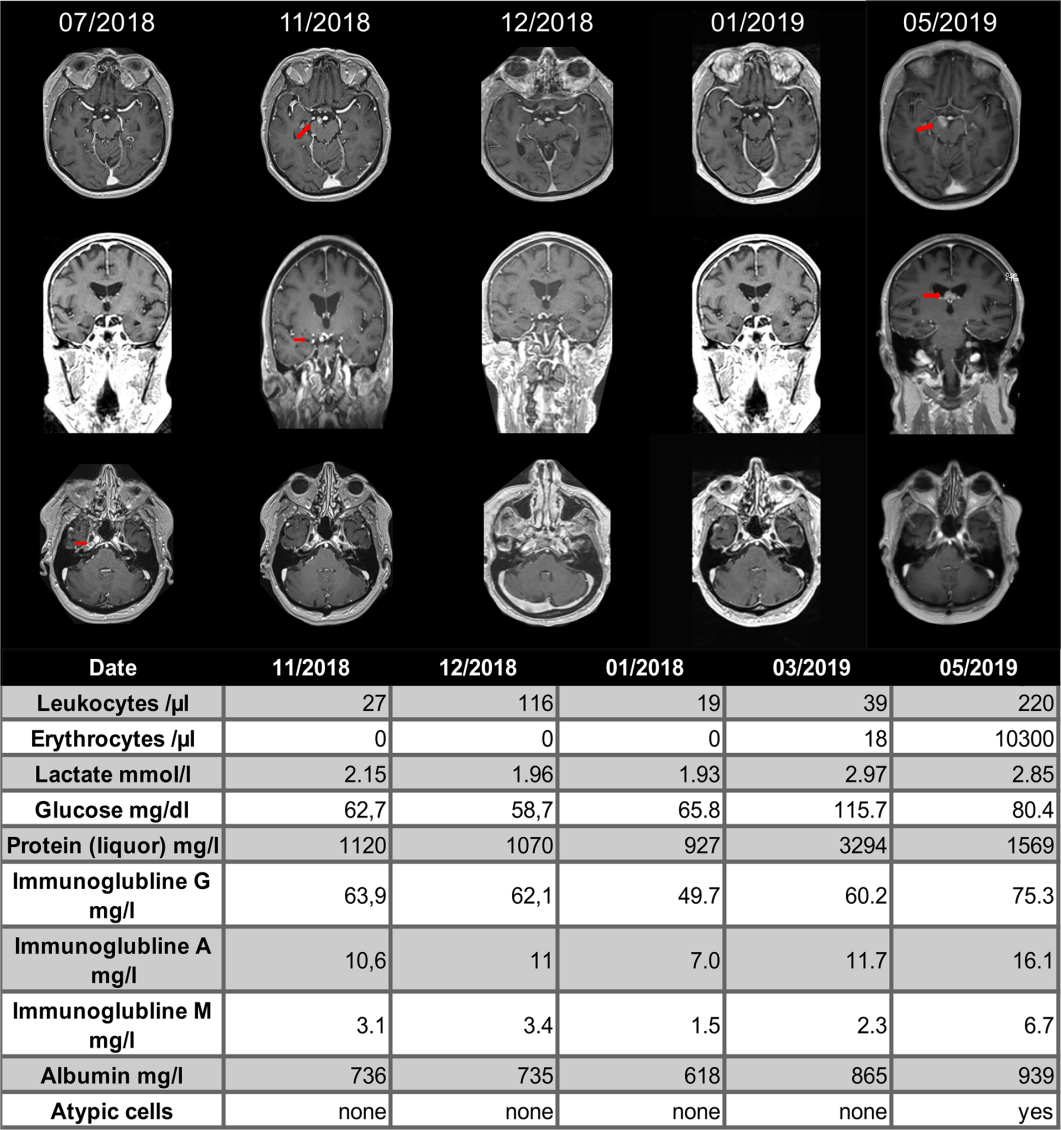
Cranial MRI scans of a 76-year-old patient with melanoma of the vulva. The patient had been treated with nivolumab and ipilimumab, which had been discontinued due to autoimmune hepatitis and switched to pembrolizumab in 03/2018. Routine staging revealed a new, contrast-enhancing tumor next to the right posterior cerebral artery (second column, red arrow). Repetitive cerebrospinal fluid analysis did not show meningeal carcinomatosis. Treatment with oral prednisolone was started in 12/2018 (third column) and the next MRI one month later showed shrinkage of the tumor, which was retrospectively diagnosed as pseudoprogression (fourth column). In 05/2019 the patient showed a meningeosis carcinomatosa in before normal appearing localizations.

As an example, in one NSCLC patient a cerebellar metastasis had been treated with stereotactic radiation (3x9 Gy) in addition to treatment with pembrolizumab. The MRI showed an excellent response of the known cerebellar metastasis to the radiation therapy, but also showed a new, distant contrast enhancement in the left frontal lobe that did not correspond to a typical image of brain metastasis (**Figure 2**). The cerebrospinal fluid (CSF) showed no significant findings (leukocytes 4/nl; erythrocytes 0/nl; lactate 1.8 mmol/l; glucose 86 mg/dl). A patient with recurrent Hodgkin’s lymphoma had been treated with pembrolizumab and had not received any cerebral radiation therapy in the past. The patient was admitted with dizziness and nausea. MRI showed small nodular, sulcal contrast enhancement as well as small bleedings in the susceptibility-weighted imaging (SWI; **Figure 3**). CSF (leukocytes 2/nl; erythrocytes 0/nl; lactate 1.98 mmol/l; glucose 52.6 mg/dl) and cerebral biopsy were not indicative of cerebral lymphoma or bacterial/viral encephalitis. The patient had been treated with high-dose methylprednisolone and a tapering dose of prednisolone in combination with everolimus, which led to an improvement of symptoms with regressive contrast enhancement in the follow-up scans. 8 months later, the patients had systemic progression and died shortly afterwards. In another melanoma patient with contrast enhancement of the cranial nerves with pembrolizumab therapy, repetitive CSF analyses were again neither indicative for meningeosis carcinomatosa nor bacterial/viral encephalitis. The enhancement disappeared during treatment with prednisolone (**Figure 4**), so the patient was diagnosed with pseudoprogression. However, in the 6-month follow-up, meningeosis carcinomatosa was diagnosed with leptomeningeal enhancement in the MRI and malignant cells in the CSF cytology (**Figure 4**). ICI treatment might therefore have “unmasked” early meningeosis by causing inflammation and subsequent disruption of the blood-brain-barrier with contrast agent enhancement (12).

### T2 Predominant ICI Therapy-Mediated Cerebral Pseudoprogression

**Figure 5:**
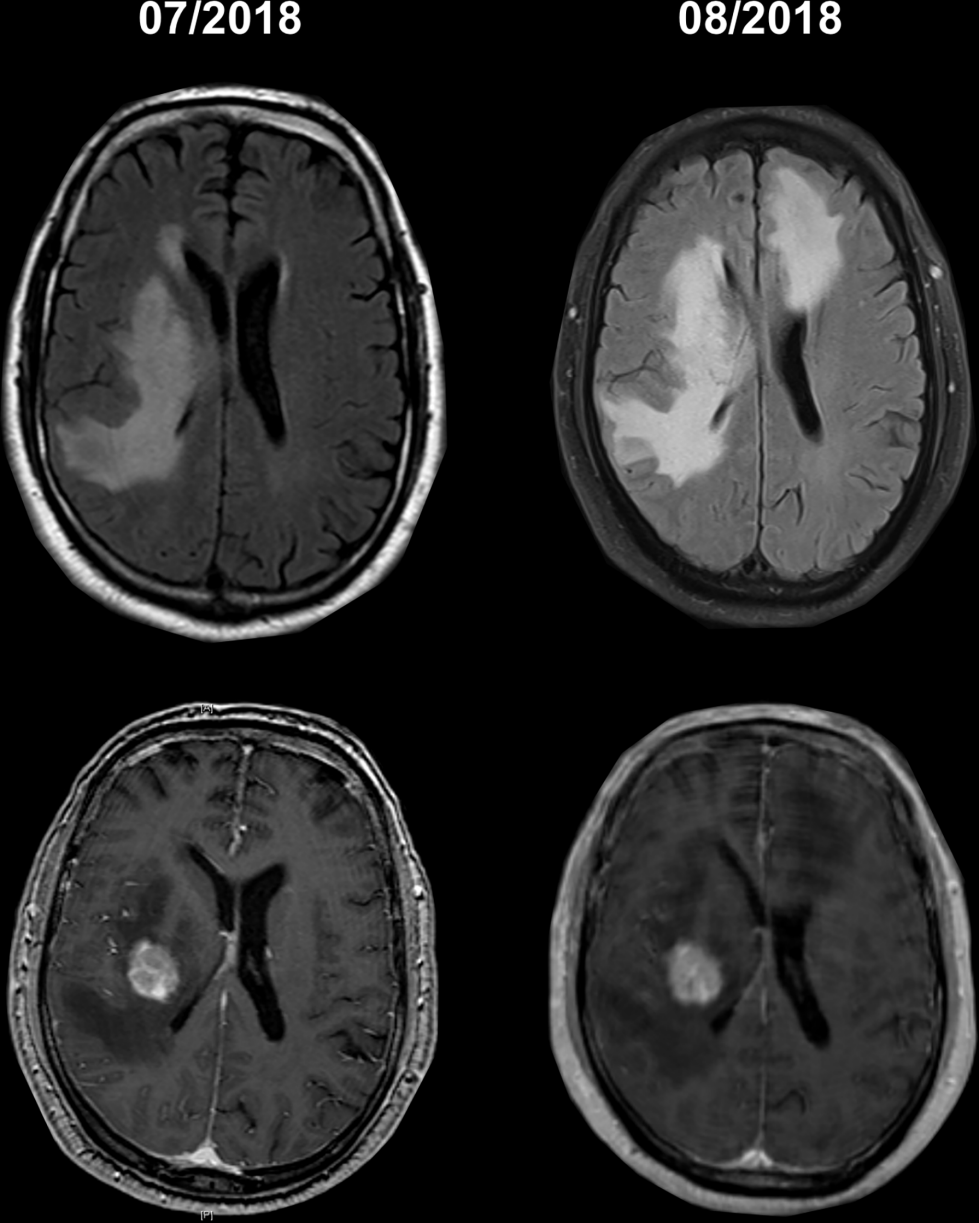
Cranial MRI scans of a 64-years-old with a metastasis malignant melanoma. The patient had been treated with radiosurgery of one metastasis (second row with contrast agent) and nivolumab after the radiation. After clinical deterioration within 4 weeks of starting checkpoint therapy, the patient showed a marked increase in T2 changes outside the radiation field.

In our collective, a pronounced increase in hyperintense lesions in T2-weighted images without contrast enhancement was observed in 5 patients during ICI therapy. In 3 cases, the T2-changes occurred next to an existing metastasis, in the other 2 cases there was no evidence of tumor infiltration in the MRI. In these 2 cases, however, the T2-weighted changes were in the former radiation field and developed rapidly after the start of checkpoint therapy (**Figure 5**). At this point, immunotherapy might significantly accelerate and amplify the phenomenon of edema due to radiogenic vascular damage or nervous demyelination through the inflammatory response.

### Vasculitis-Like Pattern of Immunotherapy-Mediated Cerebral Pseudoprogression and Discrimination From Vasculitis

**Figure 6:**
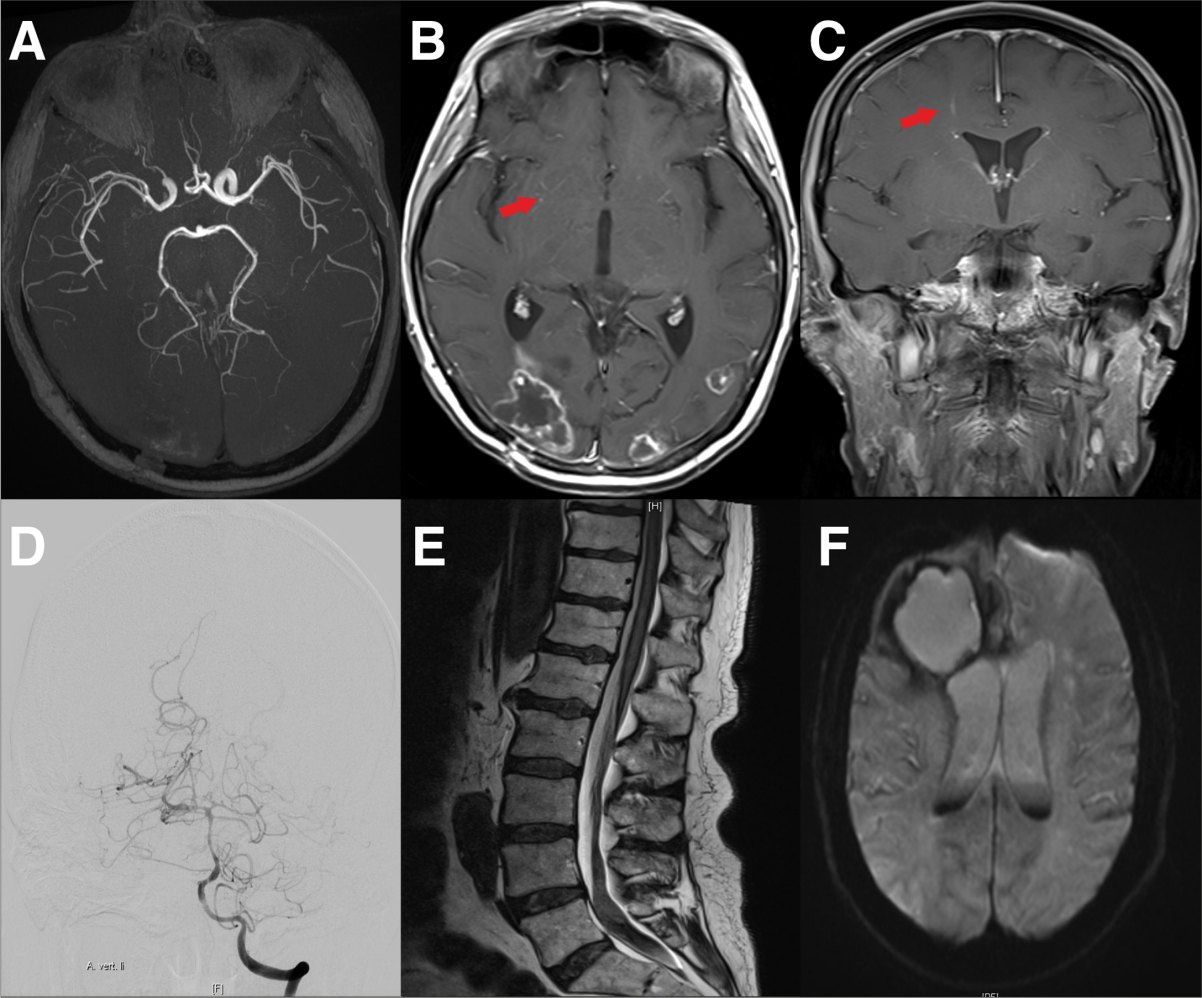
Cranial MRI of a 44-year-old female patient with NSCLC and pembrolizumab therapy. **(A)** Shows vascular imaging with no evidence of vasculitis-type changes in the large cerebral vessels. **(B)** Transversal gadolinium enhanced T1 weighted MRI with the remains of the occipitally located brain metastasis, as well as periventricular contrast medium accumulations suspicious for vasculitis (arrow). **(C)** A frontal gadolinium enhanced T1 weighted MR of the same patient. Again, the arrow indicates suspicious contrast agent accumulations. **(D–F)** Cranial MRI Scans and vertebral column MRI of a 72-year-old patient with melanoma. The Patient had been treated with nivolumab for 15 months. The patient then developed a headache and paraparesis. Cerebral angiography showed caliber changes of the left middle cerebral artery and the basilar artery. Due to vasculitis, the patient developed prolonged bleeding with siderosis-associated myelopathy. **(D)** Cerebral angiography with caliber changes of the cerebral vessels. **(E)** Cranial MRI of the lower spinal cord with bleeding in the caudal region. **(F)** Cranial MRI with bleeding of the metastasis and blood in the liquor system.

In 2 of the patients a vasculitis-like pattern was found. In one patient with brain metastases caused by NSCLC, we observed a diffuse perivascular/vascular contrast enhancement in the basal ganglia accompanied by diffusion restrictions in the same areas. The first patient showed no neurological symptoms. An increased cell count in the CSF was found 2 months after the start of therapy with pembrolizumab, but CSF showed no malignant cells or elevated lactate (leukocytes 8/nl; erythrocytes 0/nl; lactate 2.14 mmol/l; glucose 58.4 mg/dl). On the one hand, these MRI findings could be explained by tumor progression in the form of a diffuse leptomeningeal metastatic spread. However, due to the absence of brain metastasis in the further course, as well as due to the absence of cancer cells in the CSF, this explanation seems unlikely. On the other hand, autoimmune small vessel vasculitis was considered due to the simultaneous diffusion restrictions. As expected, the basal large cerebral arteries were normal in the time-of-flight MR angiography TOF, and flow rate in color duplex sonography was not increased (**Figures 6A–C**).

The second patient with brain metastases from malignant melanoma presented with headache and decreased vigilance. The cMRI showed new small cerebral DWI spots in the short-term course of a week. In contrast to the previous case, cerebral angiography and MR angiography showed a segmental narrowing of the cerebral vessels and no improvement with initial prednisolone therapy. Therefore, we assumed the development of acute cerebral vasculitis in this patient. In addition, the patient developed cerebral hemorrhage from a metastatic vessel. The hemorrhage was connected to the subarachnoid space and resulted in spinal siderosis with 4364300/nl erythrocytes in the CSF analysis (leukocytes 4572/nl; erythrocytes 4364300/nl; lactate 7.4 mmol/l; glucose 30.9 mg/dl) and consecutive paraparesis. Thus, angiography is essential in the presence of vasculitis-suspect lesions in the brain under checkpoint therapy to differentiate between the vasculitis-like pattern of immunotherapy-mediated cerebral pseudoprogression and actual vasculitis (**Figures 6D–F**).

### Therapy and Outcome

Upon diagnosis of pseudoprogression, ICI treatment was discontinued in all cases with neurological symptoms and additional steroid treatment was initiated. Six patients received dexamethasone with an initial dose of 12 mg/d and subsequent slow tapering. 3 patients received a monotherapy with 60-100 mg prednisolone for 4 days or 250-1000 mg methylprednisolone for 3 days. In addition to prednisolone, 1 patient received an immunosuppressive therapy with the anti-TNFα antibody infliximab and another patient received additional therapy with everolimus. Although more than 80% of the patients showed a decrease in neurological symptoms afterwards, the median survival after initial diagnosis of pseudoprogression was limited to only 4 months (range 0-13 months). A single patient dropped out of follow-up and 2 patients are still alive at the date of submission (**Table 2**).

**Table 2:** Outcome.

**Number of deaths**	75% (9)
**Cause of death**	
n/n	8.3% (1)
pseudoprogression	50% (6)
underlying disease	16.7% (2)

## Discussion

Immune-checkpoint inhibitors (ICI) are one of the most important clinical advances for a wide range of malignancies, including melanoma and lung cancer - diseases that frequently metastasize to the brain. Although treatment with ICIs is common in patients with brain metastases, there are no systematic evaluations of cerebral pseudoprogression, and the incidence of this phenomenon is still unknown (10, 21). The screening of all patients treated with ICIs who received sufficient brain MRIs in our institution showed an incidence for cerebral pseudoprogression of approximately 9.8% during treatment.

The phenomenon of pseudoprogression during immuno-therapy was recognized in trials for melanoma patients first and led to the update of the RANO criteria (18). The iRANO criteria (2016) assist to differentiate between pseudoprogression and progressive disease on cMRI scans (19). The iRANO criteria propose that immunotherapy can be continued despite progressive disease in MRI when (1) the new lesion or progression of a known lesion manifests within six months of immunotherapy initiation and (2) there are no new or significantly worsened resulting neurological deficits. Strict adherence to the second criterion would mandate classification of all but one of our cases (**Table 1**) as progressive disease. However, it can be assumed that regardless of the underlying pathophysiology, any brain lesion is suitable to cause clinical deficits. Therefore, we believe that the severity of clinical symptoms should be taken into account when deciding to either continue an (otherwise) effective ICI treatment or to discontinue ICI treatment and start immunosuppressive therapy. However, to discontinue the treatment would leave the tumor untreated or at least potentially undertreated. We propose a classification containing two criteria to stratify the clinical significance of cerebral pseudoprogression (**Table 3**).

**Table 3:** Pseudoprogression classification.

Category	Localization	Clinical symptoms	Therapy
**1a**	Increase of known tumor target lesion or manifestation/unmasking of prior unknown tumor	Non-significant	Continue ICI
**1b**		Significant	Try supportive medication, Stop ICI if no improvement
**2a**	Distant lesion without underlying tumor manifestation	Non-significant	Continue ICI
**2b**		Significant	Stop ICI

Side effects and symptoms should be considered “significant” if they correspond to CTCAE grade 3 and 4 or if they correspond to an CTCAE grade 2 that does not resolve with supportive therapy.

The first criterion pertains to the localization of the suspicious lesion (category 1). The pseudoprogression can be on target, which means the change in MRI can be an asymptomatic (1a) or symptomatic (1b) epiphenomenon of the desired immune response to the tumor. An existing tumor can expand in diameter and develop increased edema as immune cells invade the tumor body and cause inflammation. In the literature, the biopsy of a pseudoprogression of melanoma brain metastasis after treatment with pembrolizumab showed hemorrhage, reactive astrocytosis, microglial cells and only a few CD8^+^ T cells (10). It may also be possible that a cerebral metastasis was already present before the start of ICI therapy, but too small to become apparent in the MRI. Administration of immunotherapy might then lead to local inflammatory reaction with increased permeability of the blood-brain barrier, making it possible for the lesion to be detected. Similar effects of an increase in vascular permeability through immunotherapies have already been observed in other diseases, such as amyloid-related imaging abnormalities (ARIA) in Alzheimer’s dementia (22). The pre-existing tumor manifestation could be “unmasked” by the immune reaction as proposed in the patient displayed in **Figure 4** (12). In this scenario, the pseudoprogression would be an indicator for a desired response to the ICI treatment.

Secondly, the suspicious lesion could be caused by an autoimmune inflammation of the brain without the presence of cerebral tumor cells (category 2). We suspect this mechanism in the patient shown in **Figures 2** and **3**, since biopsy of the lesion did not reveal any signs of lymphoma cells. The sole autoimmune nature of the cerebral lesions in such cases is in accordance with the mechanism of checkpoint inhibitor-mediated hepatitis, where acute inflammatory reactions are present without evidence of tumor cells or toxic necrosis (23).

The second criterion is the severity of clinical deterioration with regard to the need for discontinuation of ICI and/or administration of immunosuppressive therapy. An asymptomatic on-target increase of contrast enhancement (category 1a) is indicative of an effective ICI therapy that should be continued. Symptomatic on-target pseudoprogression (category 1b) requires careful evaluation of whether discontinuation of ICIs is necessary. If possible, symptomatic treatments for adverse events CTCAE grade 1-2 should be optimized first. Off-target asymptomatic pseudoprogression by autoimmune mimicry (category 2a) is an adverse event and should be monitored closely when the tumor is otherwise responding to therapy (24). Symptomatic off-target pseudoprogression (category 2b), especially CTCAE grade 3-4, should be treated decisively.

In contrast to several previous case reports which describe pseudoprogression as an indicator of a good response to immunotherapy, the majority of our patients showed severe clinical symptoms and treatment had to be terminated in 11 out of 12 cases, with 6 patients dying shortly after the diagnosis of cerebral pseudoprogression (**Table 2**). This overall high morbidity is based on 2 different mechanisms. On the one hand, a recurrence of tumor after the necessity to end immunotherapy which could be observed in 2 patients. A much larger proportion of patients died before progression could occur (only a few days to weeks after the onset of neurological symptoms). In these patients (n=6), it can be assumed that the severe neurological symptoms and their subsequent effects led to death.

The pattern described in this article is morphologically similar to the reaction to radiotherapy as seen on MRI. This reaction, which is also termed as radionecrosis (25), could be exaggerated by combination with ICIs, because radiotherapy disrupts the blood-brain barrier and allows immune cells to migrate into the central nervous system in a higher number (26). Radiotherapy also increases the release of tumor antigens in the extracellular space, therefore possibly further triggering an immune response (27). This desired synergistic effect might also lead to a higher rate of pseudoprogression. In metastasized melanoma to the brain, it was shown that a combination of radiotherapy and ICIs results in a higher rate of pseudoprogression than a combination of radiotherapy and targeted therapies (28).

## Limitations

One major limitation is the retrospective character of the study. Another limitation is the low rate of biopsy-confirmed pseudoprogression. Specimens for histology could only be obtained in 2 cases mainly due to the poor condition of the other patients and/or rejection of the biopsy by the patient or their legal guardians. Nevertheless, the imaging course of the lesions as well as supporting diagnostic measures (including repetitive CSF analysis or PET scans) confirm that the lesions were indeed likely to have been caused by pseudoprogression (29).

## Conclusion

In summary, ICI-mediated cerebral pseudoprogression is a diagnostic and therapeutic challenge for clinicians, and is likely to increase in frequency in the coming years as use of ICIs grows more common. In this work, we propose a system for categorization and specific handling procedures that will support informed decision making when deciding between discontinuation of an otherwise effective immunotherapy and (risk of) patient morbidity. The low incidence of asymptomatic pseudoprogression might be underestimated because cancer patients without neurological symptoms frequently do not receive MRI staging of the brain.

## Data Availability Statement

The original contributions presented in the study are included in the article/supplementary material. Further inquiries can be directed to the corresponding author.

## Ethics Statement

Ethics approval for this retrospective data collection was obtained from the ethics committee of the University Hospital Frankfurt; Goethe University, Germany (Protocol-Number: SNO-13-2019). Written informed consent for participation was not required for this study in accordance with the national legislation and the institutional requirements.

## Author Contributions

HU: Design and conceptualized study; acquired and analyzed the data; drafted the initial manuscript for intellectual content. ES: Acquired and analyzed the data; drafted the initial manuscript for intellectual content. EH: Acquired and analyzed the data. KF: Major role in the acquisition of data. MM: Major role in the acquisition of data. MS: Major role in the acquisition of data. AK: Major role in the acquisition of data, revised the manuscript for intellectual content. AS: Interpreted the data; revised the manuscript for intellectual content. M-TF: Major role in the acquisition of data. PB: Major role in the acquisition of data. MR: Interpreted the data; revised the manuscript for intellectual content. JS: Interpreted the data; revised the manuscript for intellectual content. MV: Design and conceptualized study; acquired and analyzed the data; drafted the initial manuscript for intellectual content. All authors contributed to the article and approved the submitted version.

## Funding

The Senckenberg Institute of Neurooncology is supported by the Senckenberg Foundation. H.U. has received funding by the Frankfurt Research Funding (FFF) program ‘Nachwuchswissenschaftler’, KF has received funding by the Frankfurt Research Funding (FFF) program ‘Nachwuchswissenschaftler’ and the ‘Clinician Scientist Program’ by the Mildred-Scheel Foundation. ES has received funding by the ‘Clinician Scientist Program’ by the Else-Kröner Fresenius Stiftung. MR has received fellowships from the UCT Frankfurt and funding from the Frankfurt Research Funding (FFF) Clinician Scientists Program.

## Conflict of Interest

PB received travel grants from Roche and Zimmer Biomet. AS reports honoraria or research funding from Arvelle Therapeutics, Desitin Arzneimittel, Eisai, GW Pharmaceuticals companies, Marinus Pharmaceuticals, UCB, UNEEG medical, and Zogenix. JS has received honoraria for lectures or advisory board participation or consulting or travel grants from Abbvie, Roche, Novocure, Medac, Med-Update and UCB. MR has received a research grant from UCB. MS has received grants and personal fees from Roche, BMS, and AstraZeneca; personal fees from Abbvie, Takeda, MSD, Pfizer, Boehringer Ingelheim, Celgene, Biontech, CureVac, Novartis, Janssen, and Tesaro.

The remaining authors declare that the research was conducted in the absence of any commercial or financial relationships that could be construed as a potential conflict of interest.

## Publisher’s Note

All claims expressed in this article are solely those of the authors and do not necessarily represent those of their affiliated organizations, or those of the publisher, the editors and the reviewers. Any product that may be evaluated in this article, or claim that may be made by its manufacturer, is not guaranteed or endorsed by the publisher.
